# Drug dosage adjustment in hospitalized patients with renal impairment at Tikur Anbessa specialized hospital, Addis Ababa, Ethiopia

**DOI:** 10.1186/s12882-015-0155-9

**Published:** 2015-10-07

**Authors:** Henok Getachew, Yewondwossen Tadesse, Workineh Shibeshi

**Affiliations:** Department of Clinical Pharmacy, School of Pharmacy, College of Medical and Health Science, University of Gondar, Gondar, Ethiopia; Department of Internal Medicine, School of Medicine, College of Health Sciences, Addis Ababa University, Addis Ababa, Ethiopia; Department of Pharmacology and Clinical Pharmacy, School of Pharmacy, College of Health Sciences, Addis Ababa University, P.O. Box 9086, Addis Ababa, Ethiopia

**Keywords:** Dose adjustment, Renal impairment, Prospective

## Abstract

**Background:**

Dose adjustment for certain drugs is required in patients with reduced renal function to avoid toxicity as many drugs are eliminated by the kidneys. The aim of this study was to assess whether appropriate dosage adjustments were made in hospitalized patients with renal impairment.

**Methods:**

A prospective cross-sectional study was carried out in the internal medicine wards of Tikur Anbessa Specialized Hospital. All patients with creatinine clearance ≤59 ml/min admitted to hospital between April and July, 2013 were included in the analysis. Data regarding serum creatinine level, age, sex and prescribed drugs and their dosage was collected from the patients' medical records. Serum creatinine level ≥1.2 mg/dL was used as a cutoff point in pre-selection of patients. The estimated creatinine clearance was calculated using the Cockcroft- Gault (CG) equation. Guideline for Drug prescribing in renal failure provided by the American College of Physicians was used as the standard for dose adjustment.

**Results:**

Nine percent (73/810) of medical admissions were found to have renal impairment (CrCl ≤ 59 ml/min). There were 372 prescription entries for 73 patients with renal impairment. Dose adjustment was required in 31 % (115/372) of prescription entries and fifty eight (51 %) prescription entries requiring dose adjustment were found to be inappropriate. Of 73 patients, 54 patient received ≥1 drug that required dose adjustment (median 2; range 1–6). Fifteen (28 %) patients had all of their drugs appropriately adjusted while twenty two (41 %) patients had some drugs appropriately adjusted, and seventeen (31 %) of patients had no drugs appropriately adjusted. No patients were documented to have received dialysis.

**Conclusion:**

The findings indicate that dosing errors were common among hospitalized patients with renal impairment. Improving the quality of drug prescription in patients with renal impairment could be of importance for improving the quality of care.

## Background

The metabolism and excretion of many drugs and their pharmacologically active metabolites depend on normal renal function. In patients with kidney dysfunction, the renal excretion of parent drug and its metabolites will be impaired leading to their excessive accumulation in the body [1]. In addition, the plasma protein binding of drugs may be significantly reduced, which in turn could influence the pharmacokinetic processes of distribution and elimination. The activity of several drug-metabolizing enzymes and drug transporters has been shown to be impaired in chronic renal failure [2].

Medication dosing errors are the most important drug-related problems in patients with renal impairment [3, 4]. Inappropriate dosing in patients with kidney disease can cause toxicity or ineffective therapy [5]. In particular, older patients are at a higher risk of developing advanced disease and related adverse events caused by age related decline in renal function and the use of multiple medications to treat co-morbid conditions [6]. Drug accumulation and toxicity can develop rapidly if dosages are not adjusted in patients with impaired renal function. Drug elimination by the kidneys correlates with the glomerular filtration rate (GFR). It is thus logical to use eGFR or eCrCl for adjusting dosages in patients with renal failure [1].

Drug dosing in renal insufficiency needs to be individualized whenever possible to optimize therapeutic outcomes and to minimize toxicity. The two major approaches are either to lengthen the interval between doses or to reduce the dose. Occasionally both interval and dose adjustments are needed [7]. Drug dosage adjustment for patients with acute or chronic kidney disease is an accepted standard of practice, though there are no clear parameters to adjust drug dosing in acute kidney injury.

The challenge is how to accurately estimate a patient’s kidney function in both acute and chronic kidney disease [8], which includes renal replacement therapy which is totally different, any Scr based equations are not valid in patients with acute kidney injury and end stage kidney disease.

Many renal function estimation approaches have been proposed, amongst which the Cockcroft-Gault (CG) equation, provides an estimate of creatinine clearance (CrCl) [9]. An apparently minor increase in serum creatinine (SCr) can reflect a marked fall in GFR. For this reason the estimation of GFR through the calculation of CrCl or eGFR using validated formula is mandatory in every patient [10]. When in doubt, appropriate information for dosing guidelines should be sought in recently published monographs or texts [11]. There are no published reports on studies that evaluate drug dosage adjustment in renal patients in Ethiopia. Therefore, this study was initiated to assess drug dosage adjustment among hospitalized patients with renal impairment at Tikur Anbessa Specialized Hospital.

## Methods

### Study area

The study was conducted in the internal medicine wards of Tikur Anbessa specialized Hospital (TASH), the largest tertiary care teaching hospital of Addis Ababa University in Ethiopia. The Hospital has about 600 beds and provides diagnosis and treatment for 370,000–400,000 clients/year.

### Study design

The study design was prospective cross sectional study involving chart review and patient interview.

### Inclusion and exclusion criteria

The source population was all patients visiting the Internal medicine department of TASH, and the study population was all inpatients in the internal medicine wards with renal impairment. Patients older or equal to eighteen years of age, patients receiving at least one pharmacological agent, hospitalized for at least one day and patients who had at least one estimated creatinine clearance value of 59 ml/min or less were included in the study. Patients not receiving any pharmacological agent, female patients who were pregnant and patients with CrCl > 60 ml/min were excluded from the study.

### Sample size determination

All patients admitted in the 4 months from April 2013 to July 2013 were considered for sampling purpose. From 810 admissions, only 73 patients were included in the final analysis based on the inclusion criteria.

### Data collection procedures

Data were collected by four ward nurses who were trained for 2 days on the extraction of data from patient files and techniques of data collection and supervised by the principal investigator to check completeness every day. Patient chart review was used to collect individual patient data including age, sex, serum creatinine (this was later used to estimate CrCL), blood urea nitrogen, co-morbid condition, reason for admission, medications prescribed during hospitalization and medications that need dose adjustment using data abstraction format. Actual weight was recorded and for those who were critical and immovable patients, either the patient, if conscious, or the care giver was asked to provide the most recent weight of the patient. We didn’t use ideal body weight unless patient’s BMI was greater than 30 kg/m^2^.

The glomerular filtration rate was estimated based on creatinine clearance from serum creatinine (SCr) using the Cockcroft Gault equation as shown below for men and women respectively:

$$ \mathrm{Men}:\ \mathrm{CrCl}\ \left(\mathrm{ml}/ \min \right) = \frac{\left[\left(140\hbox{-} \mathrm{age}\right) \times \mathrm{weight}\ \left(\mathrm{kg}\right)\right]}{\mathrm{SCr}\ \left(\mathrm{mg}/\mathrm{dl}\right) \times 72} $$

$$ \mathrm{Women}:\ \mathrm{C}\mathrm{r}\mathrm{C}\mathrm{l}\ \left(\mathrm{ml}/ \min \right) = \frac{\left[\left(140\hbox{-} \mathrm{age}\right) \times \mathrm{weight}\ \left(\mathrm{kg}\right)\right] \times 0.85}{\left(\mathrm{S}\mathrm{C}\mathrm{r}\ \left(\mathrm{mg}/\mathrm{dl}\right) \times 72\right)} $$

Serum creatinine concentrations were measured using the two-point, fixed-time kinetic Jaffé reaction on a Humalyzer 3000 automated analyzer (HUMAN Gesellschaft für Biochemica und Diagnostica mbH, Wiesbaden, Germany). The serum creatinine results were not calibrated using isotope–dilution mass spectrometry (IDMS) method.

SCr level ≥1.2 mg/dL was used as a cut-off point in the pre-selection rather than CrCl due to several reasons; first SCr values was available in the patients’ medical files, however, neither body weight nor CrCl was available in the patients’ medical files. SCr value was the only laboratory value available for the physician in the patients’ medical files. So using SCr values reflected the current situation in the hospital. The SCr value of 1.2 mg/dL was considered the upper normal value for SCr in clinical practice [12]. Appropriateness was determined by comparing practice with the guideline “Drug Prescribing in Renal Failure: Dosing Guidelines for Adults and Children (Aronoff et al., 2007)” [13].

### Operational definitions

*Appropriat****e*:** when the drug regimen is adjusted based on the patient’s CrCl as recommended by the guideline “Drug Prescribing in Renal Failure: Dosing Guidelines for Adults and Children” [13].

*Inappropriate*: when the dosage prescribed is not in conformity to the patient’s CrCl as recommended by “Drug Prescribing in Renal Failure: Dosing Guidelines for Adults and Children” [13].

*Hospitalized patient*: A patient admitted in Hospital at least for 24 h.

*Prescription entries*: Lines of prescriptions in which a certain medication may be prescribed on multiple occasions for different patients.

*Renal impairment*: is a medical condition in which the kidneys fail to adequately filter waste products from the blood.

*Renal related*: is a condition where the primary diagnosis is one or another type of kidney injury

Stage of renal impairment- is the severity of renal impairment based on CrCl value regardless of the definite cause of CKD.

### Ethical clearance

Letter of ethical clearance was obtained from the School of Pharmacy Research Ethics review Board and the Department of Internal medicine Research and ethics committee, School of medicine, College of Health Sciences, Addis Ababa University. Additionally informed verbal consent for participation in the study was obtained from all participants.

### Data analysis

Data were edited, cleaned and analyzed using Statistical Package for Social Science (SPSS) version 17. The data were summarized and described using tables and graphs. Univariate and multivariate analysis were performed to compute crude odds ratio (COR) and adjusted odds ratio (AOR). Statistical significance was set at *p* value < 0.05.

## Results

As shown in Table 1, a total of 810 patients with SCr ≥1.2 mg/dL were identified during the 4-months study period between April and July 2013. Based on the inclusion criteria, a total of 73 patients (9 % of medical admissions) were included in the final analysis. Those 73 patients were designated as the renal impairment group which consisted of 40 (55 %) males and 33 (45 %) females. The median age of patients with renal impairment was 42 years (range 18–87); the median weight of patients was 60 kg; 18 (25 %) patients were admitted due to renal related disease and no attempt was made to make a distinction between CKD and AKI. Comorbidity was present in 62 (85 %) of patients among 73 renal impaired patients.

**Table 1:** Demographic and clinical data of patients with renal impairment in Tikur Anbessa specialized hospital, Addis Ababa, Ethiopia, August 2013

Demographic and clinical data	Number (%)
Total number of hospitalized patients during the study period	810
Number of Patients with renal impairment	73 (9 %)
Male	40 (55 %)
Female	33 (45 %)
Age (median)	42 (range 18–87)
SCr (mean)	2.24 ± 1.5
Estimated CrCl (mean)	39.6 ± 1.4
Drugs per patient (mean) ± SD	5.1 ± 2.3
Drugs required dose adjustment per patient (mean) ± SD	1.6 ± 1.3
Patients with stage of renal Impairment	
Stage 3	53/73 (72.5)
Stage 4	15/73 (20.5)
Stage 5	5/73 (7)
Reason for admission	
Renal related	18 (25)
Non- Renal	55 (75)
Comorbidity	
Present	62/73 (85)
Absent	11/73 (15)

The median number of drugs prescribed per patient was 5 (range 1–12) and 40/73 (54.8 %) had ≥5 drugs prescribed. The mean estimated CrCl was 39.6 ml/min (IQR 29.8–49.2), with a mean SCr valueof 2.24 mg/dl (IQR 1.3–2.3). No patients were documented to have received dialysis when the prescription was reviewed for dose adjustment.

**Fig. 1:**
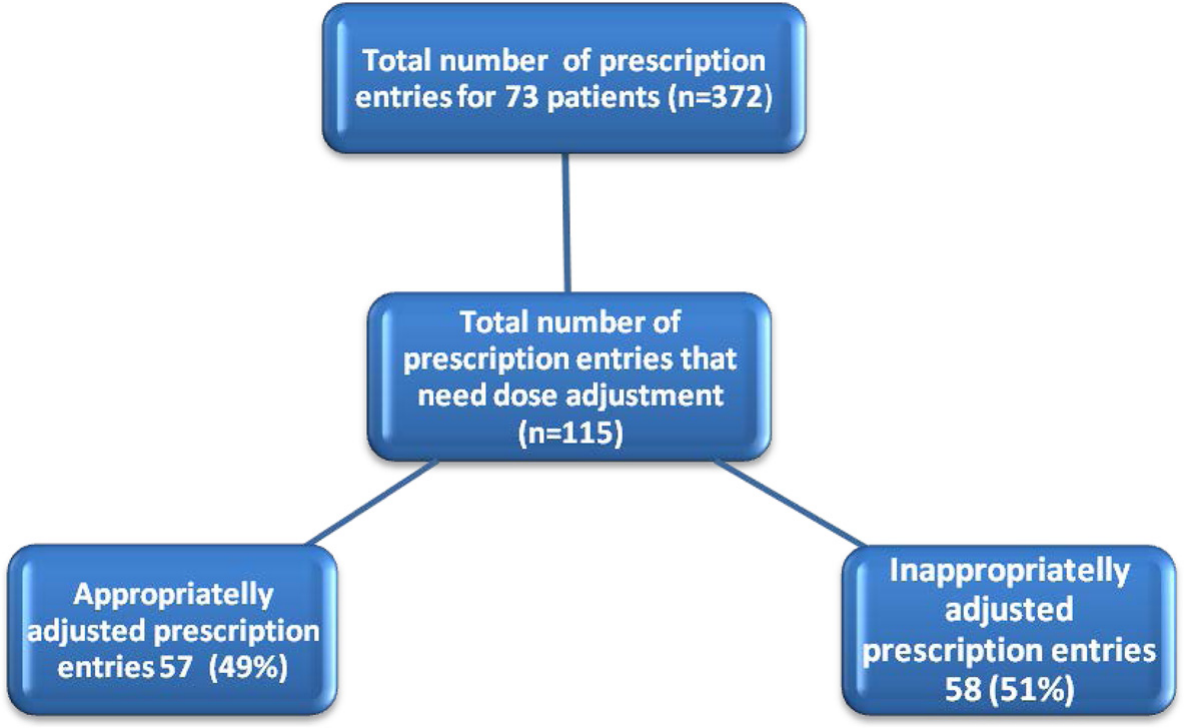
Appropriateness of prescription entries in all patients (*n* = 73) of the study, Tikur Anbessa Specialized Hospital, Addis Ababa, Ethiopia, August 2013

**Fig. 2:**
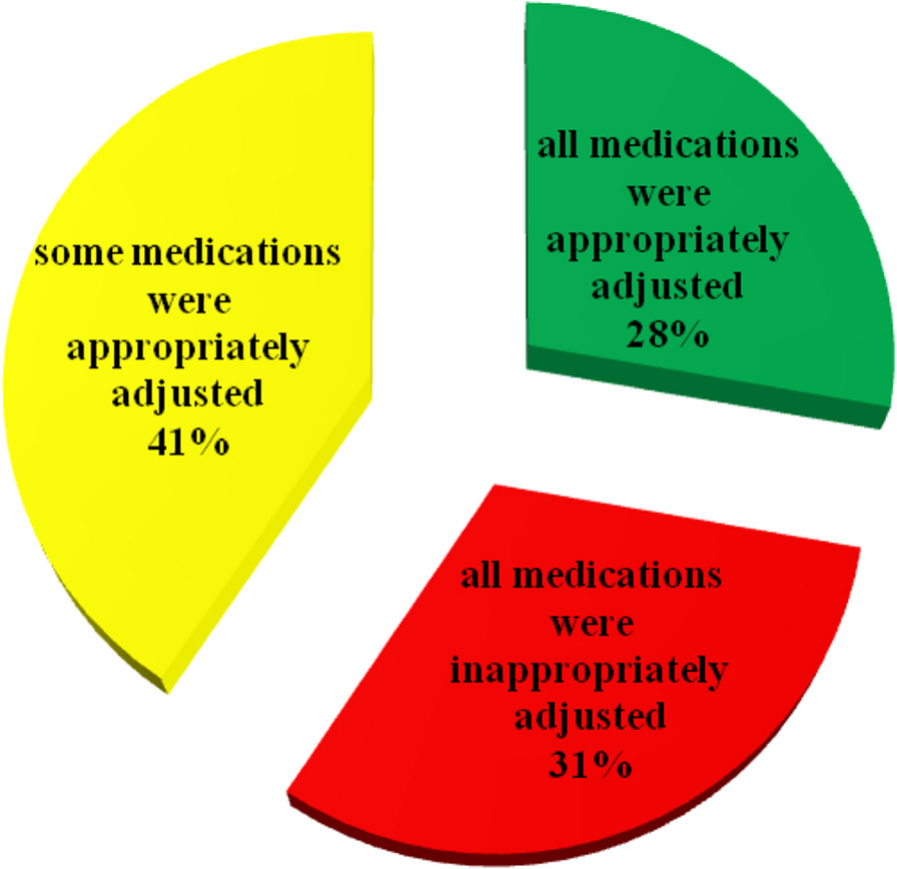
Proportion of appropriately adjusted prescription entries per patient at Tikur Anbessa Specialized Hospital, Addis Ababa, Ethiopia, August 2013

**Fig. 3:**
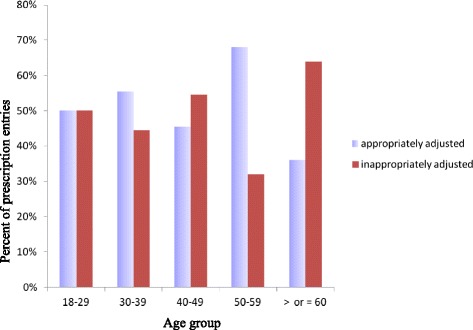
Dose adjustment of prescription entries across various age groups at Tikur Anbessa Specialized Hospital, Addis Ababa, Ethiopia, August 2013

Dose adjustment was required in 115 (31 %) of 372 prescription entries. Of the 115 prescription entries, 58 (51 %) were found to be inappropriate (Fig. 1). Analysis of the proportion of appropriately adjusted prescription entries per patient indicated that of the 73 patients, 54 (74 %) received ≥1 drug that required dose adjustment (median 2; range 1–6). Patients who had all of their medications appropriately adjusted were 15 (28 %); 22 (41 %) of patients had some drugs appropriately adjusted whereas 17/54 (31 %) of patients had all drugs inappropriately adjusted (Fig. 2). Age related analysis of dose adjustment indicated that a greater proportion of inappropriate dose adjustment in prescription entries was observed in the elderly (≥60 age group) (Fig. 3).

**Fig. 4:**
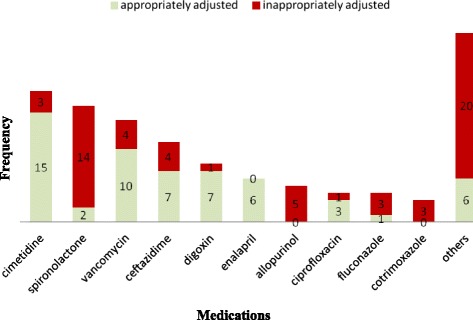
Dose adjustment by types of medication at Tikur Anbessa Specialized Hospital, Addis Ababa, Ethiopia, August 2013

When type of medication and dose adjustment were evaluated, cimetidine was the most frequently prescribed drug that required dose adjustment, which was appropriately adjusted in 15/18 (83.3 %) of cases; followed by spironolactone, vancomycin and ceftazidime which were appropriately adjusted in 2/16 (12.5 %), 10/14 (71.4 %) and 7/11 (63.6 %) of cases respectively. Enalapril was the only drug correctly dose adjusted in all cases (6/6). Allopurinol and co-trimoxazole remained unadjusted in all cases. Medications that were less frequently prescribed (≤2) were categorized under “others” (Fig. 4).

**Fig. 5:**
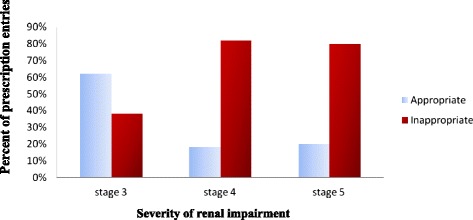
Dose adjustment of prescription entries by stage of renal impairment at Tikur Anbessa Specialized Hospital, Addis Ababa, Ethiopia, August 2013

Based on the stage of renal impairment, data showed that a total of 83/115(72 %) prescription entries that required dose adjustment were prescribed to patients with stage 3. Of 83 prescription entries, 51 (61.4 %) were appropriately adjusted for patients with stage 3. Of the 22 prescription entries, 4 (18.2 %) were appropriately adjusted for patients with stage 4. Patients in stage 5 had a total of 10 prescription entries of which 2 (20 %) were appropriately adjusted (Fig. 5). In the present study, few medications were inappropriately prescribed in stage 5 renal impairment. Two ceftazidime, one cimetidine, one vancomicin, one fluconazole and others (three) were inappropriately adjusted in patients with stage 5 renal impairment.

On univariate and multivariate analysis, COR and AOR revealed that age, sex, weight, SCr, CrCl, BUN, reason of admission, comorbidity, stage of renal impairment, number of medication prescribed per patient and number of medications that required dose adjustment per patient did not show significant difference on the proportion ofappropriately adjusted prescriptions per patient (Table 2).

**Table 2:** Relationship between independent variables and proportion of appropriately adjusted prescription entries per patient in Tikur Anbessa specialized hospital, Addis Ababa, Ethiopia, August 2013

Variables	All medications per patient were inappropriately adjusted		*P*-value	OR (95 %) CI	
	Yes	No		COR	AOR
Sex					
Male	10	19		1.00	1.00
Female	7	18	0.446	0.74 (0.23,2.36)	0.35 (0.04,3.35)
Age					
18-29	6	9	0.281	1.00	1.00
30-39	1	4	0.551	0.56 (0.11,3.02)	0.59 (0.03,14.21)
40-49	4	6	0.424	1.50 (0.12,19.44)	2.92 (0.06,127.78)
50-59	3	10	0.667	0.56 (0.09,3.52)	0.42 (0.023,7.677)
≥60	3	8	0.043	1.25 (0.196,7.96)	0.58 (0.03,10.10)
Stage					
Stage 3	8	31	-	1.00	1.00
Stage 4	7	4	0.99	3.88 (0.471,31.91)	0.73 (0.00,715.2)
Stage 5	2	2	0.99	0.57 (0.06,5.78)	0.10 (0.00,20.32)
Weight (mean)	62.88	59.59	0.221	0.98 (0.93,1.03)	1.03 (0.92,1.16)
SCr (mean)	3.27	1.88	0.898	0.51 (0.31,0.84)^a^	0.19 (0.03,1.25)
BUN (mean)	103.56	80.38	0.053	0.99 (0.98,1.00)	1.03 (0.99,1.06)
Reason of admission					
Renal	7	7	0.45	1.00	1.00
Non renal	10	30		0.33(0.09,1.18)	0.89 (0.08,10.03)
Comorbidity					
Present	14	32	-	1.00	1.00
Absent	3	5	0.109	1.37 (0.29,6.55)	0.31 (0.02,5.29)
No of Med prescribed Per patient (mean)	4.41	6.03	0.234	1.39 (1.04,1.89)^a^	1.39 (0.89,2.19)
-No of Med need Dose adjustment per patient (mean)	1.76	2.30	0.17	1.75 (0.89,3.46)	1.57(0.54,4.55)

^a^statistically *significant; COD* crude odds ratio; *AOR* adjusted odds ratio

**Table 3:** Relationship between independent variables and appropriateness of dose adjustment of prescription entries in Tikur Anbessa specialized hospital, Addis Ababa, Ethiopia, August 2013

Variables	Appropriately adjusted		*P*-value	OR (95 %) CI	
	Yes	No		COR	AOR
Drugs prescribed					
Cimetidine	15	3	0.00	0.060 (0.013, 0.280)	0.013 (0.001, 0.15)^a^
Spironolactone	2	14	0.24	2.100 (0.369, 11.96)	4.639 (0.353, 60.919)
Vancomycin	10	4	0.013	0.120 (0.027, 0.525)	0.045 (0.004, 0.525)^a^
Ceftazidime	7	4	0.04	0.171 (0.037, 0.792)	0.067 (0.005, 0.894)^a^
Digoxin	7	1	0.008	0.043 (0 .004, 0.421)	0.009 (0.000, 0.297)^a^
Enalapril	6	0	0.998	0.000 (0.000, −)	0.000 (0.000, −)
Allopurinol	0	5	0.999	4.846 (0 .000, −)	1.365 (0.000, −)
Ciprofloxacin	3	1	0.055	0.100 (0.009, 1.147)	0.017 (0.000, 1.091)
Fluconazole	1	3	0.969	0.90(0 .078, 10.327)	1.071 (0.033, 34.663)
Cotrimoxazole	0	3	0.99	4.846 (0.000,-)	4.686 (0.000, −)
Other	6	20	-	1	1
Stage					
Stage 3	51	32	0.016	0.157 (0.031, 0.786)	64.159 (0.159, 2.59)
Stage 4	4	18	0.174	1.125 (0.170 7.452)	587.70 (4.040, 8.549)^a^
Stage 5	2	8	0.012	1	1
SCr (mean)	1.83	2.83	0.02	1.820 (1.232, 2.690)	129.95 (6.431, 2.626)^a^
BUN (mean)	85.2	100.4	0.027	1.004 (0.998, 1.010)	0.976 (0.956, 0.997)^a^
Reason of admission					
Renal	10	18	0.684	2.115 (0.877, 5.10)	0.612 (0.057, 6.534)
Non renal	47	40	-	1	1
Comorbidity					
Yes	50	53	0.098	1.484 (0.442, 4.98)	11.77 (0.635, 218.265)
No	7	5	-	1	1

^a^Statistically significant, *COD* crude odds ratio; *AOR* adjusted odds ratio

However, dose adjustment of prescription entries was associated with type of medications prescribed, stage of renal impairment, SCr level and BUN (Table 3). There was a negative association between the type of medication prescribed and the likelihood of appropriately adjusting medications. When cimetidine (AOR = 0.013 (0.001, 0.150)), vancomycin (AOR = 0.045 (0.004, 0.525)), ceftazidime (AOR = 0.067 (0.005, 0.894)) and digoxin (AOR = 0.009 (0.000, 0.297)) were prescribed, dose was appropriately adjusted less frequently than any other medications. Prescription entries were appropriately adjusted more frequently in stage 4 than any other stages of renal impairment (AOR = 587.70 (4.040, 8.549)). Specifically each 1-unit increase in SCr level was associated to an increase in the likelihood of appropriately adjusting dose of medications by a factor of 129.95.

## Discussion

The present study evaluated the drug dosage adjustment in hospitalized patients with renal impairment. The prevalence of renal impairment was 9 % of internal medicine ward admissions. Comparing this result to Decloedt et al. (32 %) study [14], the figure is low.

This may be attributed to the fact that we used a serum creatinine cutoff point, rather than eGFR to define renal impairment. It is likely that we may have missed some patients with renal impairment as a result of this.

This study also assessed the proportion of appropriately adjusted drugs per patient. In this study, 74 % received ≥1 drug that required dose adjustment. That means 26 % of patients did not have any medications that required dose adjustment. These patients might have been prescribed medications that either did not require dose adjustment or nephrotoxic medications might have been avoided or switched to safer drugs. Among those who received medications that required dose adjustment, fifteen (28 %) patients had all of their drugs appropriately adjusted; twenty two (41 %) patients had some drugs appropriately adjusted and seventeen (31 %) patients had no drugs appropriately adjusted. In Decloedt et al. [14] study, 71 % received ≥1 drug that required dose adjustment. All drugs were correctly adjusted in only 12 % of patients; some drugs were correctly adjusted in 29 % of patients and no drug was correctly adjusted in 59 % of patients. This study suggested that a lot has to be done yet regarding dose adjustment. However, the findings were much better than a similar study done in South Africa [14].

The total number of prescription entries that required dose adjustment and the percentage of appropriate dosing varied in different studies [14–17]. Our study has different figures of prescription entries that required dose adjustment (31 %) when compared with other studies, Decloedt et al. (19 %), Sweileh et al. (19 %) and Salomon et al. (71 %) [14–16]. The doses were found to be inappropriately high in 42.2 % [17]. Appropriate dosing in this study (49 %) was much higher than Decloedt et al. (32 %), Sweileh et al. (26.42 %) and Salomon et al. (34 %) studies [14–16].

In this study, the percentage of appropriately adjusted prescription entries was higher than the findings in South Africa [14] and Palestine [15]. This is quite encouraging but may not be surprising as TASH is the largest teaching hospital in the country with many specialists and residents in training that are supposed to have better awareness of dose adjustment compared to physicians in general hospitals. However, the figure of this study was less than the study findings in France [16] and Australia [17]. Most developed countries have introduced an automated system of reporting renal function with eGFR which alerts physicians of the need for dose adjustment.

The other finding reported in the present study is that SCr had a positive association with appropriate prescribing. It appears that physicians become more careful in medication prescription and make appropriate dose adjustments among patients with elevated SCr. Assessment of the relationship between age and dose adjustment indicated that a higher proportion of inappropriate prescription entries was found in the age group ≥ 60. This is in keeping with the well known fact that using SCr, that underestimates the presence and degree of renal impairment in the elderly, often results in improper dose adjustment [6].

Our study has some limitations. The sample size may be considered rather small. Our study was a cross sectional study and the study design did not allow us to make a distinction between acute kidney injury and chronic kidney disease. The study, hence, cannot answer the question whether there was a need for frequent dose adjustment that may be necessary in those with rapidly changing kidney function. It is quite conceivable that in addition to consideration of renal function, prescribers may have made dose adjustments on the basis of other parameters like the blood pressure, heart rate, electrolyte levels. Physicians may have used guidelines other than the one we used in the study to make dose adjustments. Using the same cut-off point(s-creatinine > 1.2 mg/dl) for all patients would result in an underestimation of the prevalence of impaired kidney function in women and the elderly. This in turn may have led to an underestimation the proportion of patients who needed dosage adjustment.

We used the CG formula and estimated the Creatinine Clearance rather than the MDRD equation to estimate GFR for various reasons. First, although the MDRD equation is widely used to estimate GFR in many parts of the world, the equation has to be validated as a measure of the GFR in a particular population before it can be adopted for use. To our knowledge there are no studies that have validated the use of the MDRD equation in an Ethiopian population. The use of the MDRD equation for drug dosing purposes often yields higher doses than the CG equation, which many believe is a safety concern [18]. CG typically yields a more conservative estimate and indicates the need for dose adjustment more often [18]. In addition little information has been published on the performance of the MDRD equation in the elderly (age > 65 years), the obese, individuals with liver disease, and races other than Caucasian or African-American and the findings have been inconsistent [19].

Estimation of GFR from combined serum creatinine and cystatin C–based equation recently published [20]. Serum cystatin C–based GFR estimates were closely comparable to MDRD (abbreviated) estimates and an equation combining it with serum creatinine, age, sex and race yielded the best possible estimates of GFR. However, the test is neither widely used nor easily available at this time, and the experience is limited [20].

## Conclusion

This study indicates that appropriate dose adjustment was not done for patients with renal impairment by practitioners in a significant percentage of patients. This finding indicates the need for providing doctors with information and guidelines for dose adjustment in patients with renal impairment to prevent poor clinical outcome and toxicity resulting from dosing errors in patients with renal impairment.

## Abbreviations

GFR: Glomerular filtration rate
CKD: Chronic kidney disease
CrCl: Creatinine clearance
SCr: Serum creatinine
CG: Cockcroft-Gault equation
IQR: Interquartile range
COR: Crude odds ratio
AOR: Adjusted odds ratio
AKI: Acute kidney injury
BUN: Blood urea nitrogen
eGFR: Estimated glomerular filtration rate
MDRD: Modification of diet in renal disease study
